# Supplementary figures and images for: Evolution of Three-Finger Toxin Genes in Neotropical Colubrine Snakes (Colubridae)
Source: Toxins (Basel). 2023 Aug 25;15(9):523. doi: 10.3390/toxins15090523 (PMC10534312; doi:10.3390/toxins15090523)

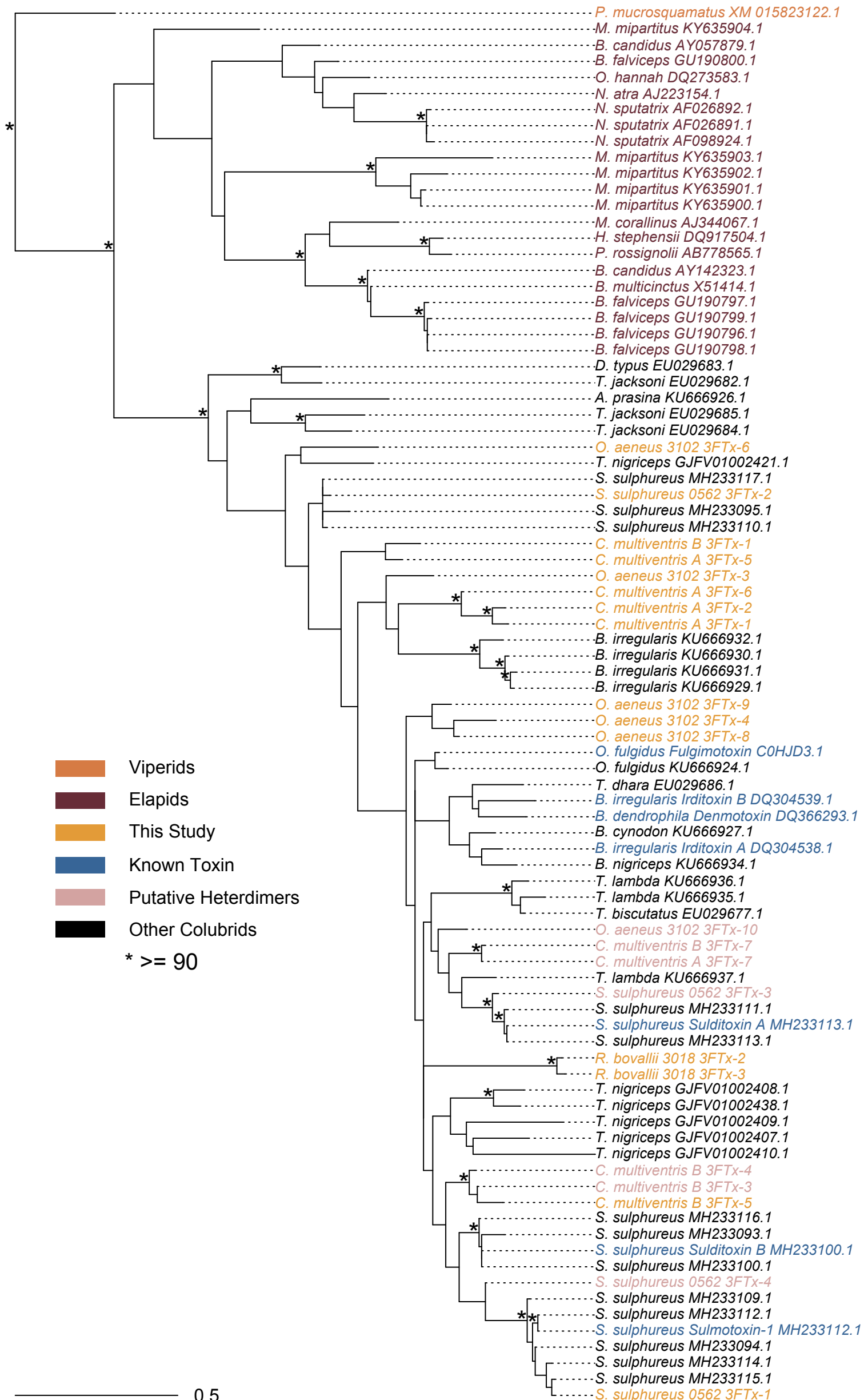

Supplement: Supplementary file 1 [file toxins-15-00523-s001.zip › Figure S1.pdf]
